# Advances of nanoparticle-mediated diagnostic and theranostic strategies for atherosclerosis

**DOI:** 10.3389/fbioe.2023.1268428

**Published:** 2023-11-09

**Authors:** Lin Lin, Lin Chen, Juan Yan, Peirong Chen, Jiahui Du, Junpeng Zhu, Xinyu Yang, Boxin Geng, Lang Li, Wen Zeng

**Affiliations:** ^1^ School of Medicine, Chongqing University, Chongqing, China; ^2^ Department of Cell Biology, Third Military Medical University, Chongqing, China; ^3^ Jinfeng Laboratory, Chongqing, China

**Keywords:** atherosclerosis diagnosis, multimodal imaging, exosome, theranostic, vulnerable plaque

## Abstract

Atherosclerotic plaque remains the primary cause of morbidity and mortality worldwide. Accurate assessment of the degree of atherosclerotic plaque is critical for predicting the risk of atherosclerotic plaque and monitoring the results after intervention. Compared with traditional technology, the imaging technologies of nanoparticles have distinct advantages and great development prospects in the identification and characterization of vulnerable atherosclerotic plaque. Here, we systematically summarize the latest advances of targeted nanoparticle approaches in the diagnosis of atherosclerotic plaque, including multimodal imaging, fluorescence imaging, photoacoustic imaging, exosome diagnosis, and highlighted the theranostic progress as a new therapeutic strategy. Finally, we discuss the major challenges that need to be addressed for future development and clinical transformation.

## 1 Introduction

Atherosclerosis (AS) is one of the most common cardiovascular disease (CVD), which is a chronic inflammatory disease driven by lipids and affects vascular wall cells. The rupture of atherosclerotic plaque will lead to a series of fatal cardiovascular events, such as myocardial infarction, stroke and pulmonary embolism (Bjorkegren and Lusis, 2022). Therefore, the diagnosis and treatment of atherosclerotic plaque is of great significance.

AS is characterized by the lifelong accumulation and transformation of lipids, smooth muscle cells (SMCs) and inflammatory cells of necrotic core within the vessel intima (Nguyen et al., 2019; Bjorkegren and Lusis, 2022). Currently, the traditional clinical diagnosis technologies for AS, such as angiography, optical coherence tomography (OCT) and intravascular ultrasound (IUVS) (Liang et al., 2018; Chow et al., 2021), can only provide the prediction of cardiovascular and cerebrovascular events by quantifying the percentage of lumen occlusion. Plaque only with severe stenosis are considered as dangerous plaque, which greatly underestimates the high-risk of mild-stenosis plaque while a large number of acute vascular events occured in mild-stenosis (stenosis<50%) (Kamtchum-Tatuene et al., 2021). The risk of vulnerable carotid plaque should be attributed not only to the degree of stenosis, but also to the plaque composition. Therefore, it has become an intense research field to accurately diagnose clinical high-risk atherosclerotic plaque through non-invasive imaging technologies. In addition, traditional AS medication prescriptions, such as statins, vasodilators, antiplatelet and anticoagulant-related thrombolytic drugs, basically work on the whole body and have obvious adverse reactions. For example, statins have hepatotoxicity and can cause rhabdomyolysis and myocardial infarction (Benes et al., 2016); Antiplatelet drugs and thrombolytic drugs have high risk of hemorrhage (Downes et al., 2019). Therefore, to improve the delivery efficiency of such drugs and achieve efficient treatment with low toxicity is also an important problem in the clinical treatment of AS. In addition, studies have shown that although statins and other existing therapies can control some manifestations of atherosclerosis, other symptoms of the disease may lead to acute events as a consequence (Nilsson, 2017). Therefore, statin therapy alone is not enough and new therapies with other mechanisms need to be developed.

Benefit from the rapid development of nanotechnology, nanoparticles have great potential applications in medical imaging (Leeper et al., 2017). Nanoparticles can adjust their physical properties and surface modification through precise control of manufacturing means. On this basis, nanoparticles can be designed as a new platform to integrate diagnostic agent and therapeutic agent for the theranostics of atherosclerotic plaque. The purpose of this review is to provide a comprehensive overview, summarizing the application of various nanoparticle-enhanced strategies in traditional imaging methods, as well as some new imaging methods such as photoacoustic imaging and exosome diagnosis, and focusing on the development of new therapeutic strategies for integrated diagnosis and treatment. Finally, we discussed the challenges and prospects of nanoparticle-based diagnosis and treatment in the field of AS plaque.

## 2 Pathological development of atherosclerosis

In the process of atherosclerosis, the dysfunction of endothelial cells (ECs) is an important pathophysiological factor leading to atherosclerosis. Activated ECs express a variety of adhesion molecules such as VCAM-1 and chemotactic molecules such as MCP-1. These molecules together promote the recruitment and accumulation of monocytes and lymphocytes (Zhu et al., 2018). Monocytes will differentiate into macrophages in subendothelial space and further polarize into proinflammatory phenotype (Nguyen et al., 2019). These proinflammatory macrophages internalize oxidized the low-density lipoprotein (oxLDL) through scavenger receptors. The increased uptake of oxLDL and the decreased efflux of cholesterol lead to lipid imbalance of macrophages and promote the formation of foam cells (Libby, 2021), the accumulation of foam cells will lead to the expansion of necrotic cores, increasing the burden and vulnerability of plaque. In addition, neovascularization is another important feature of the vulnerable plaque, which will further promote the recruitment of proinflammatory monocytes and lipoprotein deposition, and increase the risk of plaque hemorrhage (Camare et al., 2017; Camps-Renom et al., 2020). The continuous accumulation of ox-LDL and proinflammatory monocytes inevitably led to the failure of timely phagocytosis-mediated clearance of apoptotic foam cells. These secondary necrotic core indicates a more vulnerable stage of the plaque. In order to stabilize the vulnerable plaque, fibrous caps containing migrating SMCs, fibroblasts and extracellular matrix including collagen will cover the surface of necrotic cores (Libby, 2021). However, with the progress of inflammation in the plaque, the fibrous cap will become thinner, or even decompose, leading to thrombotic occlusion and other clinical events [1, 2].

**FIGURE 1:**
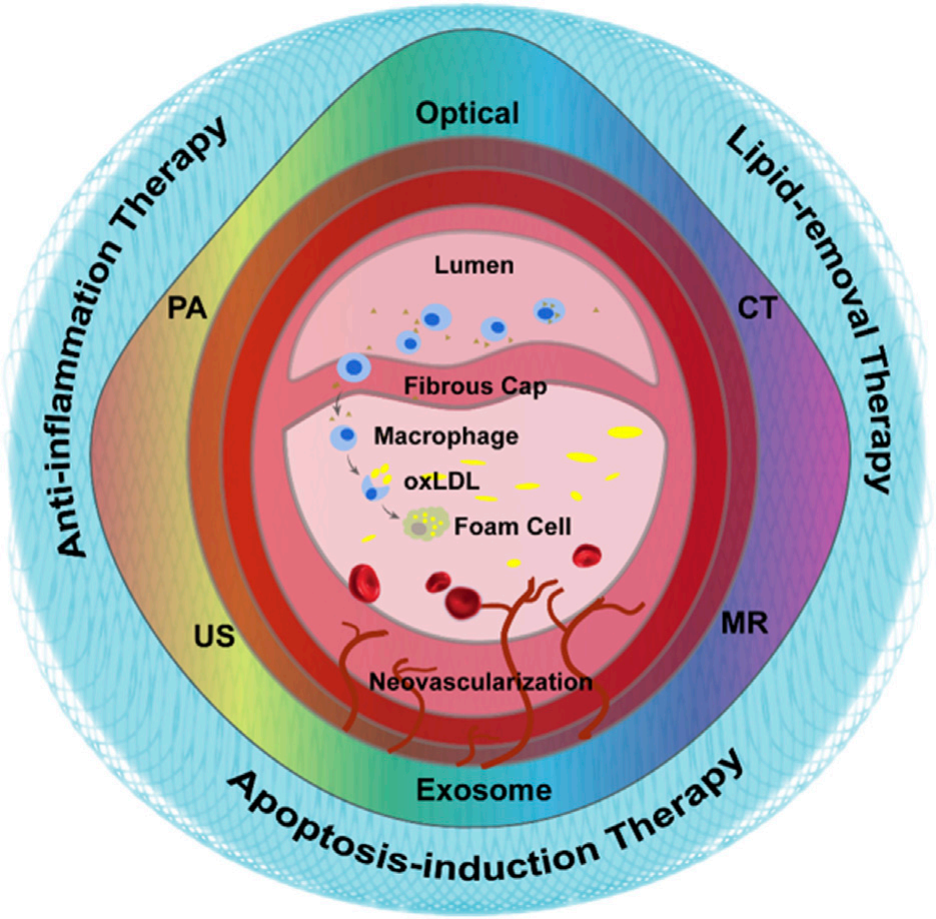
Graphic representation of the pathological development of atherosclerosis and the methods for atherosclerosis diagnosis and treatment.

The imaging of AS needs to reflect the exact plaque biology and pathophysiology specifically and effectively, including ECs activation, foam cells accumulations, inflammation, angiogenesis and thrombosis. The key processes and associated cellular events was shown in Figure 1, providing many enrichment targets for nanoparticle-assisted diagnosis and treatment of atherosclerosis.

## 3 Nano-strategies based on conventional imaging technologies

### 3.1 PET/CT

It is challenging to diagnose the non-calcified plaque with computed tomography (CT) because soft tissues are not sensitive to X-ray attenuation (Zhang et al., 2021). Positron emission tomography (PET) with 18F-FDG has been proposed as a non-invasive gold standard to identify the vulnerability of plaque (Kwiecinski, 2022), since the tracer-uptake was strongly connected with macrophage infiltration (Joshi et al., 2014). However, excessive myocardial metabolic uptake frequently resulted in erroneous signals (Chen and Dilsizian, 2013).

### 3.2 Magnetic resonance imaging (MRI)

MRI is widely used in clinic with the advantages of non-invasive, radiation-free and has a high contrast for imaging soft tissues (Bruno et al., 2019). However, it is difficult to determine the morphology of atherosclerotic lesions in the early stages while the vessels are non-obviously narrow. Meanwhile, it is difficult to distinguish the microstructures (such as the fibrous cap), due to the resolution limitations.

### 3.3 Contrast enhanced ultrasound (CEUS)

**FIGURE 2:**
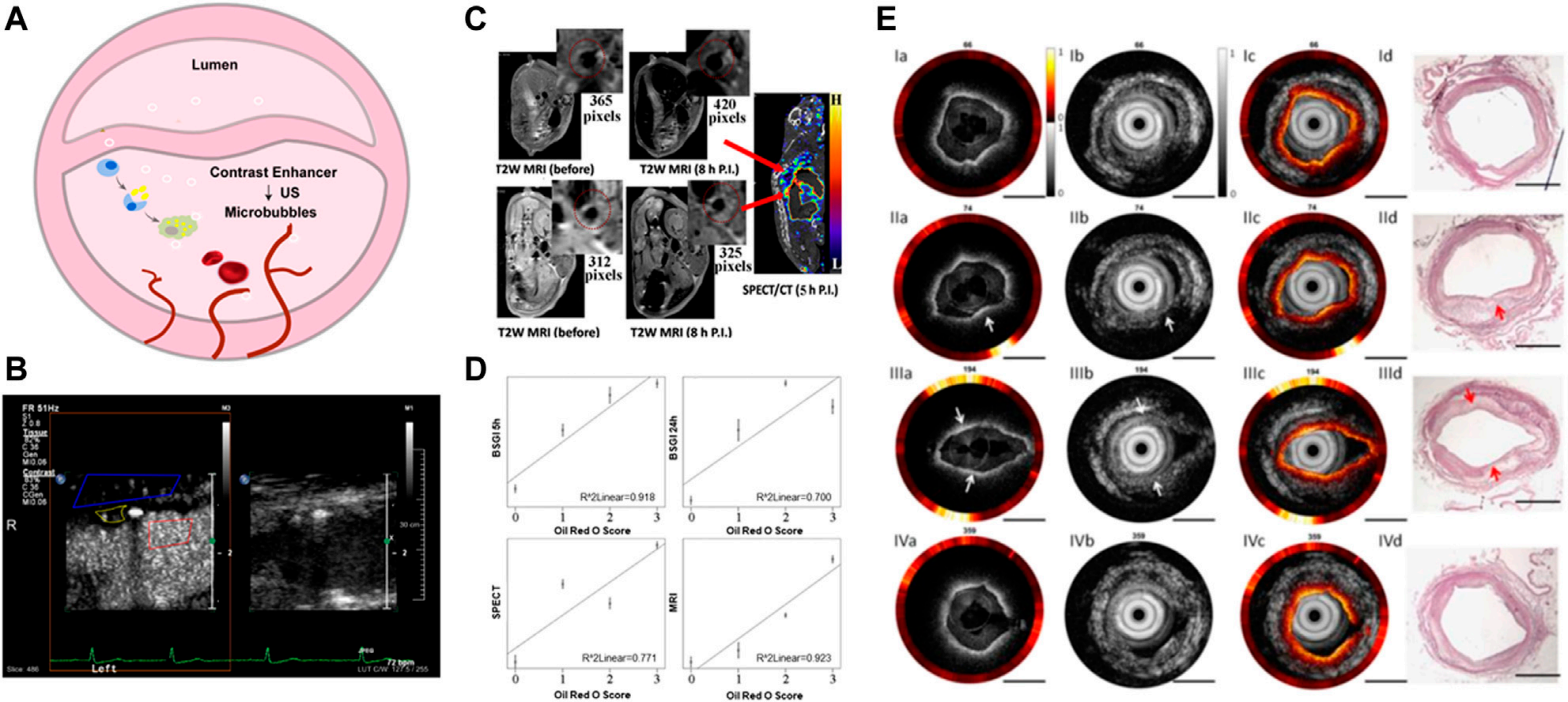
Nano-strategies based on conventional imaging technologies. **(A)** Schematic diagram of vulnerable atherosclerotic plaque assessment by targeted microbubbles. **(B)** B-Mode and CEUS imaging for the vulnerable plaque. Reprinted with permission (van den Oord et al., 2013). **(C)** SPECT/MRI images of ^99m^Tc−DTPA−USPIO−Annexin V in atherosclerotic plaque *in vivo*. **(D)** The linear relationship between diagnosis signals with pathological staining. Reprinted with permission (Cheng et al., 2015). **(E)** Tri-modality images of atherosclerotic plaque in rabbit. Scale bar = 1 mm. The Opyica Publishing Group request a certain type of citation as “Reprinted with permission (YAN and TENG, 2017) © The Optical Society.”

Contrast enhanced ultrasound (CEUS) molecular imaging is a technology that relies on ultrasound to detect molecular or cellular events occurring within blood vessels by detecting targeted microbubbles (Ten Cate, 2015; Schinkel et al., 2016; Cismaru et al., 2021) (Figure 2A). The latest EFSUMB clinical practice guidelines recommended the use of CEUS imaging to evaluate the neovascularization in plaque, so as to better stratify the risk of plaque (Figure 2B, van den Oord et al., 2013) (Dietrich et al., 2020). Early screening and risk grading of atherosclerosis can be achieved by targeting such components as JAM-A (Curaj et al., 2018), ICAM-1 (Li et al., 2021), activated vWF (Tian et al., 2021) and apoptotic macrophages (Xiaoju Ma et al., 2022) at atherosclerotic plaque.

### 3.4 Multimodal imaging

Accurate assessment of plaque is critical. Clinical study has determined the criteria for vulnerable plaque, namely, 1) large lipid pool, 2) thin fibrous cap and 3) severe inflammatory reaction (Bentzon et al., 2014). As above mentioned, each traditional imaging method is limited (Table 1). The advantages of different imaging techniques can be combined by using hybrid imaging methods to overcome the restrictions. As a result, multimodal imaging has been widely explored.

**TABLE 1:** Benefits and drawbacks of different imaging technologies.

Technical name	Principle	Advantages	Limitations	References
Computed Tomography (CT)	Different human tissues have different sensitivity to X-ray absorption and transmission	Short time, high resolution	Radiation, lack of spatial resolution and low sensitivity	Zhang et al. (2021)
Positron Emission Tomography (PET)	Radioactive tracer (18F-FDG)	High sensitivity, quantifiable	Radiation, low resolution, myocardial-background	Joshi et al. (2014)
Magnetic Resonance Imaging (MRI)	Energy level transition of atomic nucleus under magnetic field	Radiation-free, high spatial resolution, high contrast of soft tissue imaging	High cost, long time, low sensitivity	Bruno et al. (2019)
Ultrasound (US)	Doppler effect	Radiation-free, economical, convenient, real-time imaging	Low resolution	Cismaru et al. (2021)

Nanotechnology has made great progress in the molecular imaging field of AS. By combining two or more imaging patterns, multimodal imaging was demonstrated to further improve the specificity and resolution, providing complementary information from different angles. Cheng et al. developed the ^99m^Tc−DTPA−USPIO−Annexin V probe, a hybrid superparamagnetic nanoparticle based on radioactive-labeling, which can overcome the low-resolution of nuclear medicine through high-resolution structural imaging of MRI (Figure 2C). This probe has the potential to identify apoptosis, which means that it can identify the vulnerable plaque, and the strong correlation between imaging signal and immunostaining parameters made it available for volume measurement (Figure 2D) (Cheng et al., 2015). In addition to Annexin V, duramycin can also effectively target the apoptotic part of the plaque. The combining results of Oil red O(ORO) staining, TUNEL staining and HE staining showed that [^99m^Tc] dulamycin closely binded to apoptotic cells of atherosclerotic lesions, and the binding activity was significantly higher than that of [^99m^Tc] Annexin V, which can be helpful of vulnerable atherosclerotic plaque quantification (Yan Hu and Soo Choi, 2017). Similarly, Su et al. modified Fe_3_O_4_ magnetic nanoparticles with GEBP11 peptide, which has high affinity for neovascularization, to achieve PET/MR dual-mode imaging of atherosclerotic plaque (Tao Su et al., 2017). Chen et al. created an US/MR dual-detectable probe by embedding perfluorooctyl bromide (PFOB) into a superparamagnetic iron oxide (SPIO) nanoparticle, and the VEGFR-2 antibody was connected to the surface of the nanoparticle to achieve the diagnosis of vulnerable plaque by targeting the neovascular system (Hua Chen et al., 2017).

These conventional techniques are not the only options available for the arrangement and fusion for multimodal imaging of atherosclerosis. The emergence of new technologies such as fluorescence imaging, photoacoustic imaging and other technologies had provided a new research direction for multimodal imaging. Nanoparticles with dual, triple or even more modalities have been widely explored. For example, OPN is overexpressed on vulnerable atherosclerotic plaque. As a potential binding target, Qiao et al. designed an osteopontin (OPN)-specific MRI/fluorescence probe to detect the vulnerable plaque. *In vitro* cell experiments showed that the probe could specifically recognize foam cells and *in vivo* visualization of vulnerable plaque could be achieved by intravenous injection of the nanoprobe (Hongyu Qiao et al., 2017). Li et al. developed a fully integrated tri-modality intravascular imaging system, which had the ability to simultaneously obtain OCT, ultrasound and fluorescence data and display images in real time (Figure 2E). It had been verified in artery models of pigs and rabbits. The probe size was 1 mm, indicating its great clinical application potential (Yan and Teng, 2017).

## 4 Nano-strategies based on novel imaging technologies

### 4.1 Fluorescence imaging

**FIGURE 3:**
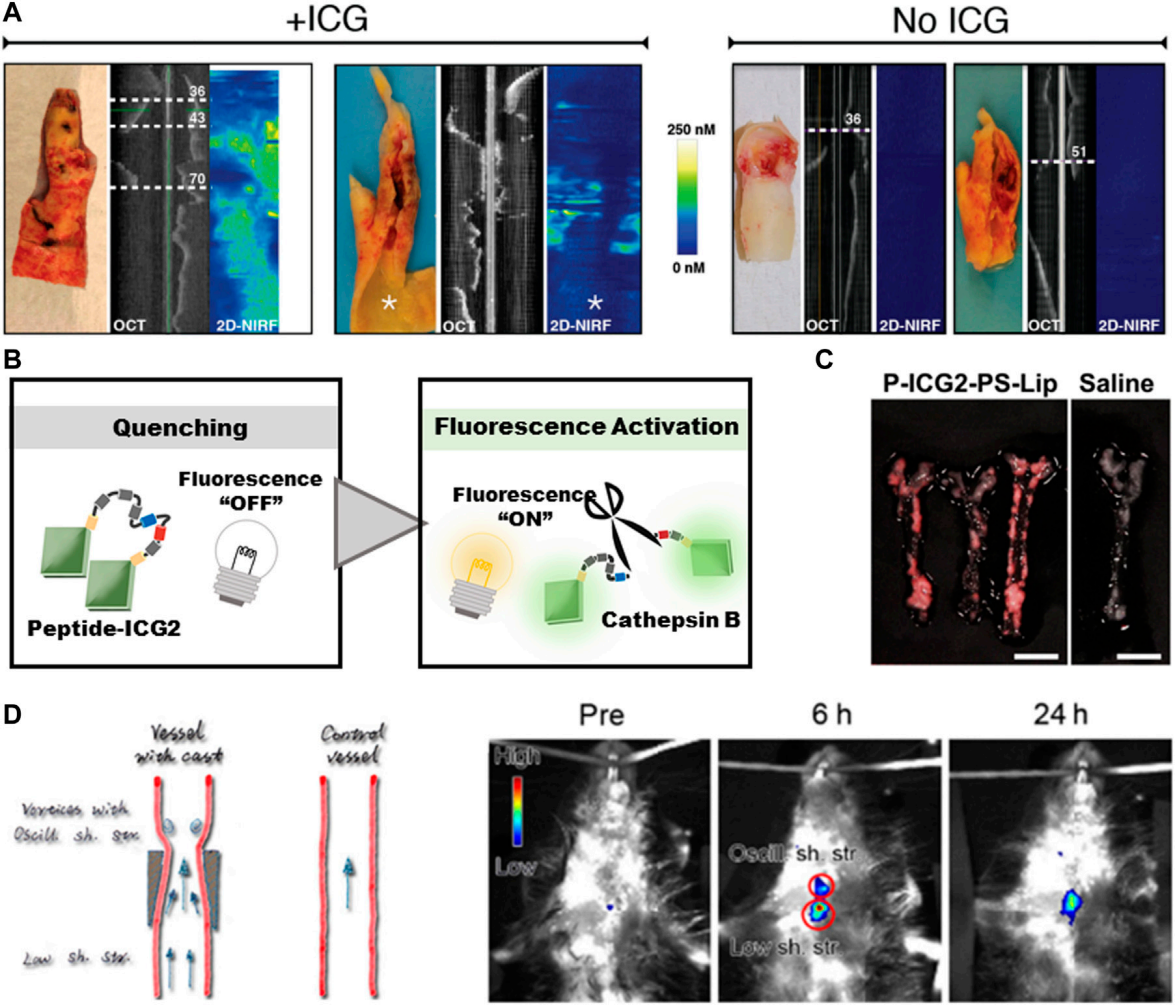
Diagnosis of atherosclerosis based on fluorescence imaging. **(A)** The signals of ICG in human carotid atherosclerosis *ex vivo*. Reprinted with permission (Johan et al., 2016). **(B)** Schematic diagram of fluorescence-switch function. **(C)** NIRF imaging of atherosclerotic plaque in ApoE^−^ mice. Scale bar = 5 mm. Reprinted with permission (Yudai Narita et al., 2019). **(D)** Upconversion luminescent images of atherosclerotic plaque captured at different time points in ApoE^−^ mice. Reprinted with permission (Qiao et al., 2017).

Near-infrared fluorescence (NIRF) is a new method for transvascular imaging (Chowdhury et al., 2022). Except the visible light range, NIRF can capture a wider range of pathological processes *in vivo*, the high tissue penetration depth and high signal-to-noise ratio make it highly sensitive. In order to achieve clinical intracoronary NIRF molecular imaging, clinical targeted NIRF imaging agents need to be provided (Hara et al., 2012). Indocyanine green (ICG, ex/em 805/830 nm) is a FDA-approved NIRF imaging probe. In 2011, a research team found that ICG may target atherosclerosis (Koivistoinen et al., 2011). By intravascular and *in vitro* NIRF imaging, ICG was found to accumulate in atherosclerotic macrophages, and was detected in atherosclerosis model of rabbits and pigs. Subsequently, in 2016, the BRIGHT-CEA trial observed the first-in-hunman ICG signals in atherosclerosis through *in vitro* intravascular NIRF-OCT and fluorescence imaging (Figure 3A), indicating the clinical application prospects of ICG molecules (Johan et al., 2016).

However, the liver can eliminate the circulating ICG rapidly, and the short half-life (2–4 min) impels the researchers to build imaging probes to prolong its lifetime (Desmettre et al., 2000). The targeting capacity of probes no only helps to prolong the circulating time, but also makes the probes fluorescence-switchable through the auto-quenching mechanism (Sano et al., 2013). In detail, the interaction between ICG molecules and carrier molecules will lead to the quenching of ICG fluorescence, and the probes can be switched from “OFF” to “ON” to emit fluorescence signals after the degradation within the targeted cells, so as to obtain a clear image by suppressing the non-specific noise signals (Figure 3B). By exploiting these properties, Since the accumulation of macrophages containing phosphatidylserine receptors is a characteristic of vulnerable plaque, Narita et al. chosen a lysosomal enzyme-cleavable peptide to silence the signals and further encapsulated Peptide-ICG2 into phosphatidylserine-enriched liposomes (P-ICG2-PS-Lip) to achieve macrophage-targeting (Figure 3C) (Yudai Narita et al., 2019). Ikeda et al. constructed an activatable fluorescence probe by conjugating the iron oxide nanoparticles (IONPs) with ICG. IONPs are biocompatible molecules with minimal toxicity and have been widely used in biomedical applications (Tran et al., 2022). The large molecular weight of IONPs enables them to accumulate in AS-associated macrophages. By increasing the imaging efficiency, the NIRF signals strongly correlated with macrophages existed in atherosclerotic plaque, thus the location and activation of macrophages can be quantitatively evaluated (Hiroyuki Ikeda and Yu, 2018).

In addition to ICG, more fluorescence probes have shown the potential for identifying the vulnerable plaque. Toll like receptor 4 (TLR4) is an important biomarker of inflammation and macrophage-transformation while foam cells play a key role in the development of vulnerable atherosclerotic plaque (Geng et al., 2021). As a TLR4-targeting fluorophore, ZW800-1C also has the ideal feature of ultralow nonspecific tissue background, another important advantage of ZW800-1C as a fluorescent agent in atherosclerotic plaque imaging is that it can be cleared by kidney within 4 h, which is different from the rapid liver-uptake of ICG (Hyun et al., 2014). Feraheme-alexa Fluor 750 is a kind of iron oxide nanoparticles, and has been approved by FDA for the treatment of iron anemia. This molecule can be recognized by proinflammatory and anti-inflammatory macrophages through scavenger receptor AI/II (Wang et al., 2019), and has been widely used, including carotid atherosclerosis. Inspired by the relatively deep tissue penetration (3–5 cm) of upconversion luminescence, Qiao et al. covalently combined foam-cell-specific OPN antibody with NaGdF4:Yb,Er@NaGdF4 upconversion nanoparticles to construct a macrophage-specific molecular probe for atherosclerosis imaging (Figure 3D) (Qiao et al., 2017). Although the number of molecular probes is increasing, NIR-II imaging is still limited by poor soft-tissue penetration, light-scattering and autofluorescence, further histological analysis is also required to verify the specificity and colocalization of NIRF probes (Hettie et al., 2020).

### 4.2 Intravascular photoacoustic (IVPA) imaging

**FIGURE 4:**
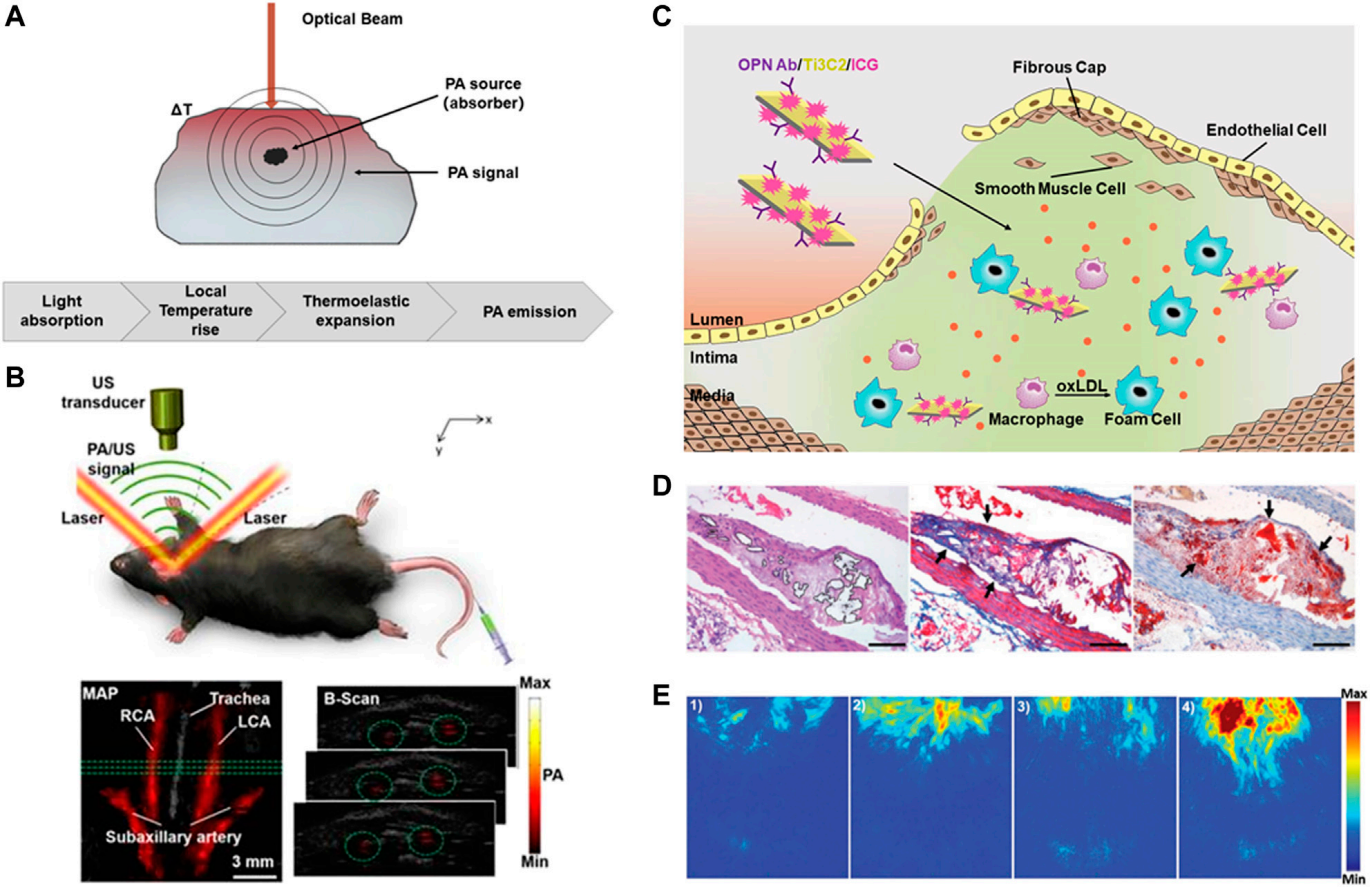
Diagnosis of atherosclerosis based on IVPA imaging. **(A)** Schematic diagram of PA imaging. **(B)** B-scan images and the PA signal in rat carotid arteries (Xie et al., 2020). **(C)** Schematic diagram of OPN Ab/Ti3C2/ICG nanoprobe. **(D)**The accumulation of OPN Ab/Ti3C2/ICG in the atherosclerotic plaque. Scale bar = 50 μm. **(E)** The PA imaging of the experimental mice *in vivo*. Reprinted with permission (Ge et al., 2020).

Intravascular photoacoustic tomography (IVPAT) is a new imaging method as a natural extension of IVUS (Li et al., 2018). As shown in Figure 4A, it uses multiple wavelengths to irradiate the selected tissues and identify the acoustic waves generated by the absorbers-mediated thermoelastic expansion to form photoacoustic images. Photoacoustic (PA) imaging combines the advantages of optical and ultrasonic modes, that is, its imaging depth are equivalent to those of ultrasonic imaging while the imaging resolution and sensitivity are as high as optical fluorescence imaging, which can conduct accurate three-dimensional (3D) imaging of deep tissues, and has the potential to detect atherosclerosis inflammation (Wang and Hu, 2012).

PA imaging can detect the molecular composition of tissues, so as to create images of tissue types. By comparing the 3D imaging of IVPAT with histological staining, Zhang et al. proved the feasibility of IVPAT to describe the spatial and quantitative characteristics of lipid-rich plaque *in vivo* (Zhang et al., 2014). In addition, after being integrated with ultrasound imaging, it can deliver morphological information and plaque size directly. In terms of intravascular applications, IVPA imaging systems with different excitation wavelengths have been reported. 461nm and 532 nm were firstly used by several research teams to image human atherosclerotic aorta (Wei et al., 2011; Li et al., 2012). The degree of lipid density in lesions is closely related to the vulnerability of plaque rupture. The most effective wavelength range for characterizing lipid components is found to be between 1,210 and 1,720 nm (Wang et al., 2012; Choi et al., 2018; Choi et al., 2019).

In addition to the definition of excitation wavelength, in the past few years, researchers have been committed to the technical improvements of IVPA technology to meet clinical requirements. For example, Cao et al. have developed a quasi collinear IVPA catheter with high sensitivity and sufficient tissue-penetration depth, and selected the sheath material with the smallest PA and US attenuation and artifact generation, which can conduct full-depth lipid localization and quantification of arterial wall (Cao and Michael Sturek, 2018). Iskander Rizak et al. used the optical resolution photoacoustic microscope (OR-PAM) system with spectral imaging function to explore lipid PA spectral signatures and its distribution in late plaque at the micro level, providing a new way of the exploration of optimal contrast of IVPA (Sophinese Iskander-Rizk and Kim Van Der Heiden, 2021). 4D-US adds the dimension of time to the three-dimensional foundation, this dynamic form can be used to quantify the altered kinematics of vascular tissues. Sangha et al. provided evidence of lipid-specific PAT signal-to-noise ratio through ORO staining, they further combined 4DUS to quantify the morphological and vascular remodeling during atherosclerosis progression (Goergen, 2020).

In addition, a variety of molecular probes have been developed to further enhance the PA signals by targeting vulnerable plaque. Wu et al. prepared a complex ICG@PEG-Ag_2_S as a PA reagent, adding amphiphilic C18 chain for lipid-targeting to achieve *in vivo* diagnosis of atherosclerosis (Chenxin Wu et al., 2016). Xie et al. used the molecular probe PBD-CD36 to label inflammatory cells and displayed inflammatory information through photoacoustic imaging, which proved the feasibility and potential of non-invasive PA molecular imaging technology to identify and evaluate carotid atherosclerosis inflammation *in vivo* (Figure 4B) (Xie et al., 2020). Gifani et al. found that single-walled carbon nanotubes (SWNTs) can be selectively absorbed by Ly-6C^hi^ monocytes, so they developed an injectable contrast agent to selectively target foam cells and their Ly-6C^hi^ monocyte precursors (Gifani et al., 2021). In addition, Ge et al. constructed OPN Ab/Ti3C2/ICG nanoprobe to target foam cells. The loading of near-infrared dye ICG further enhanced the PA signal of vulnerable plaque in the aortic arch, thus achieved *in vivo* imaging of vulnerable plaque successfully (Ge et al., 2020) (Figures 4C–E).

### 4.3 Exosome-mediated diagnosis

Exosomes is a type of extracellular vesicles with initial invagination of endosomal ranging 40–100 nm in diameter (Loyer et al., 2014; Kalluri and LeBleu, 2020). As a key medium of intercellular communication, exosomes plays an important role in the development of atherosclerosis (Wang C. et al., 2021; Zhang et al., 2022). The changed components may be an important regulator of atherosclerosis. Different from traditional diagnostic imaging methods such as US, CT and MRI, the diagnostic principle of exosome-based nanoplatform is based on the parameters of exosome-specific compositions at biological molecular level, such as the quantitative changes of nucleic acids and proteins, so as to accurately judge the biological state of donor cells, liquid biopsy based on exosomes has been proposed (Bathini et al., 2018). Among them, miRNA may play an important role in the early diagnosis of atherosclerosis due to the following advantages: it is not affected by PH; It can withstand repeated freezing and thawing; It is conservative in all species; There are many quantitative detection methods (Bathini et al., 2018; Zhang et al., 2022). Theoretically, exosome-derived miRNAs are better biomarkers than circulating plasma-derived miRNAs, because the purification can ensure the sensitivity and specificity of exosomes derived from specific cell types. Therefore the detection of miRNA changes is helpful to predict the risk of atherosclerosis.

Jiang et al. found that distinct expression of exosome-derived miRNAs, such as miR-122-5p, miR-192-5p, were new biomarkers for predicting the ischemic recurrence in intracranial atherosclerosis disease (Jiang et al., 2019). Xue et al. found that miR-221 and miR-222 were important for atherosclerosis, providing new biomarkers for clinical diagnosis of atherosclerosis (Xue et al., 2015). In addition, miR-146a, miR-21-5p, miR-208a, miR-17, miR-92a, MiR-223-3p and miR-122-5p were expressed in patients’ peripheral blood, and have become promising biomarkers for diagnosis, risk stratification and prognosis prediction (Chen et al., 2015; Huang Y. et al., 2017; Huang YQ. et al., 2017; Guo et al., 2020; Sandeep Singh et al., 2020). With more in-depth investigation, miRNA is also connected to several primary disorders in the development of atherosclerosis. For example, Zhang et al. found three diabetes-related circulating miRNAs in atherosclerosis: miR-21, miR-218, and miR-211. Their research showed that plasma miRNAs may be an effective biomarker for early detection of atherosclerosis induced by diabetes (Zhang et al., 2016). MiR-30 may be an useful biomarker of atherosclerosis in patients with essential hypertension (Huang et al., 2016). The level of miR-17 was significant in acute ischemic stroke, which may be related to stroke recurrence (Kim et al., 2015). Gao et al. believed that miR-126 and miR-143 can be used as important biomarkers of cerebral atherosclerosis (Gao et al., 2019). Although miRNAs have demonstrated good diagnostic capability, the same miRNA may has diverse activities *in vivo* depending on the absorbing-cells. For example, miR-155 can promote atherosclerosis but it can also reduce VSMC proliferation by inhibiting angiotensin-converting enzyme at the same time (Zheng et al., 2017), indicating that we need more clinical data to find significant miRNAs in order to work out the best therapeutic schedule. Like all other biomarkers, Exosomes must be verified and approved by the International Organization for standardization before being used in clinical settings.

**FIGURE 5:**
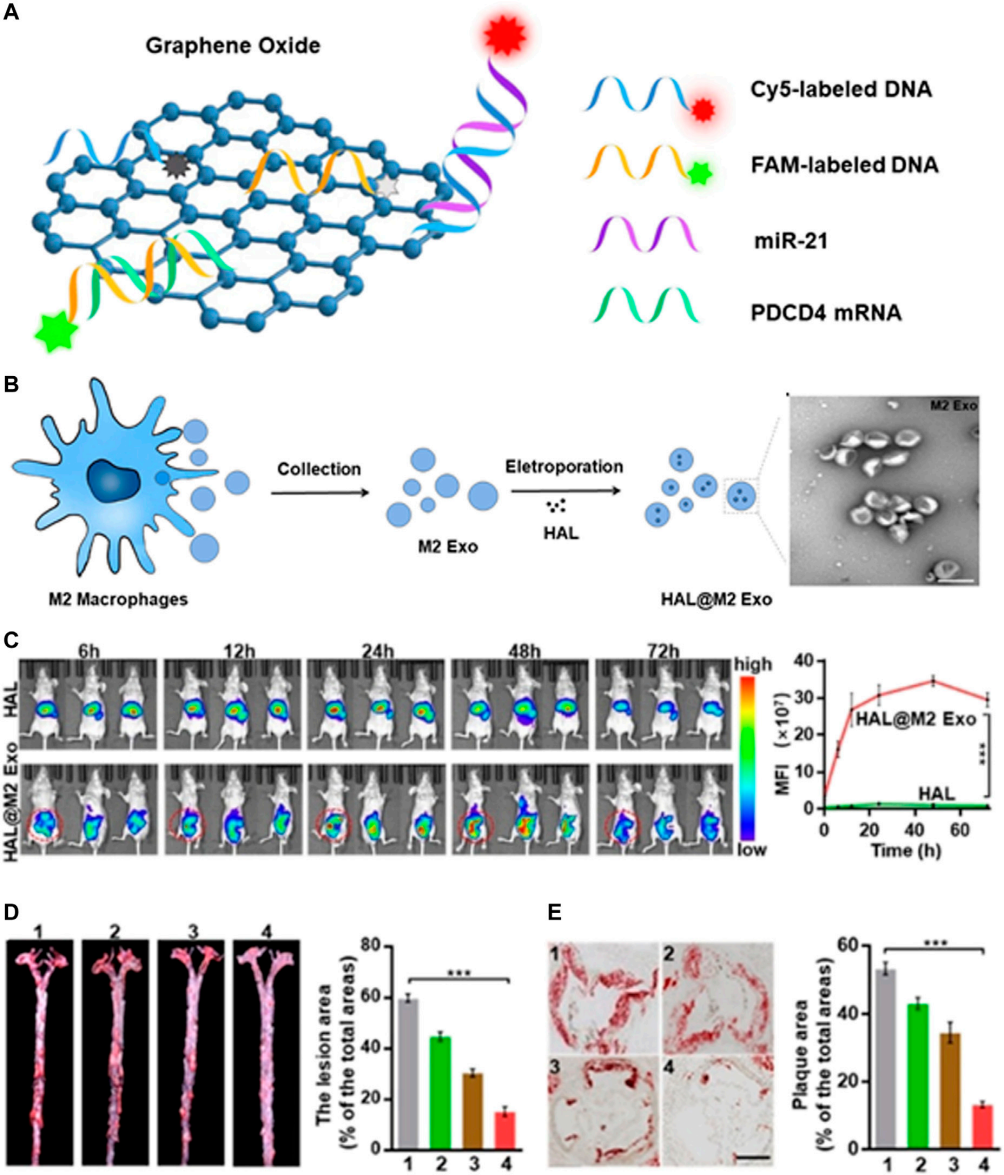
Diagnosis of atherosclerosis based on exosomes. **(A)** Schematic illustration of the nanoprobe for the detection of miR21 and PDCD4 mRNA. **(B)** Schematic illustration of the synthesis of HAL@M2 Exo. **(C)** Fluorescence imaging of HAL or HAL@M2 Exo treated mice at different time intervals. **(D)**Photographs and **(E)** HE staining of plaque and the corresponding quantitative analyses of plaque areas. Scale bar = 300 mm. Reprinted with permission (Guanghao Wu et al., 2020).

In addition to molecular diagnosis, researchers further combined fluorescence imaging to realize the visualization of miRNA in living cells. Pan et al. used graphene oxide (GO) and two different fluorescence-labelling single-strand DNA (ssDNA) to construct a miRNA sensing platform, which can achieve the monitoring and visualization of miR-21 and PDCD4 mRNA in living cells simultaneously (Figure 5A). *In vitro* experiments demonstrated that the nanoplatform showed excellent selectivity and sensitivity to miR-21 and PDCD4 mRNA. More importantly, the results of fluorescence confocal imaging and RT-PCR verified the negative correlation between miR-21 and PDCD4 mRNA, indicating that the nanoprobe had the ability to recognize RNA of different expression levels (Pan et al., 2018). Xiong et al. used P1&P2@rGO nanoprobe to realize the simultaneous two-color imaging of miR-451a and miR-214-3p in human breast cancer cells. These simple and effective strategies provided a universal sensing platform for highly sensitive detection and simultaneous imaging of double miRNAs in living cells (Xiong et al., 2021).

Not only can it serve as a diagnostic tool, exosomes can also be used for drug delivery (Fan et al., 2021; Liang et al., 2021). However, the transformation of exosomes as drug delivery carriers is hindered by their low loading efficiency. The enrichment of ligands on the surface of engineered exosomes makes it possible for receptor-mediated tissue targeting. Sun et al. established a novel adipose-specific exosome delivery strategy, encapsulated engineered mRNA activated by tissue specific microRNA (miRNA) translation, and improved the delivery efficiency through ultrasound targeted microbubble destruction (UTMD) and minimized the off-target effect (Sun et al., 2020). Wu et al. carried out molecular engineering transformation on the exosomes (M2 Exo) derived from M2 macrophages, and further electroporated them with hexyl 5-aminolevulinate hydrochloride (HAL) approved by FDA to obtain self-biosynthesizable exosomes (Figure 5B). After systematic administration, the engineered M2 Exo showed excellent anti-inflammatory effect via the chemokine receptors presented on the surface of M2 Exo. Protoporphyrin IX (PpIX), the intermediate of the heme-biosynthesis pathway, permitted fluorescent imaging of AS (Figure 5C). The encapsulated HAL can create anti-inflammatory carbon monoxide and bilirubin via its own biosynthesis and heme-metabolism, further strengthening the anti-inflammatory effect on the basis of the intrinsic anti-inflammatory properties of M2 macrophages (Figure 5D), the multifunctional nanoparticles combining diagnosis and treatment may broaden its clinical application (Guanghao Wu et al., 2020).

## 5 Nano-strategies for the integrated diagnosis and treatment of atherosclerosis

### 5.1 Anti-inflammation therapy

The construction of multifunctional nanoparticles integrated with diagnosis and treatment is conducive to clinicians’ real-time monitoring and regulation of therapy intensity to achieve precise treatment of atherosclerotic plaque. Atherosclerotic plaque is characterized by the deposition of cholesterol and macrophages, which are not only the main components of plaque, but also the key sources of inflammation. Therefore, the efficient elimination of these two elements from plaque will have substantial therapeutic advantages (Gao et al., 2020; Kim et al., 2020; Wang Y. et al., 2021).

Xu et al. reported a new type of magnetic mesoporous silicon nanoparticles FITC-VHP-Fe_3_O_4_@SiO_2_ by connecting VHPKQHR peptide (VHP) to target the highly expressed VCAM-1 on injured endothelial cells in atherosclerotic plaque. *In vivo* experiments showed that it is a good contrast agent for MRI and can be used to diagnose atherosclerotic plaque. The large specific surface area and pore volume of mesoporous silica nanoparticles provided the possibility of subsequent drug delivery (Xu et al., 2019). Still by connecting the VHP peptide, Chen et al. (Huang et al., 2022) synthesized a rapamycin (Rap)-coated magnetic liposomes (Fe_3_O_4_@VHP-Lipo) with great clinical application for the diagnosis and treatment of early atherosclerotic plaque in mice. As a result of the damaged endothelial layer, the vascular wall’s increased permeability allowed liposomes to readily penetrate the plaque after being recognized by ECs. The Rap can dissolve foam cells slowly under the acidic conditions of the plaque, lower the levels of oxidized LDL. The synthesized theranostic nanoparticle enabled the bimodal imaging of atherosclerosis through MRI and fluorescence imaging. Also, due to the improvement of targeting and the extension of circulation duration, Rap can achieve a considerable therapeutic benefit at a lower dose of 2 mg (Rap)/kg, which was much lower than what was previously reported, and side effects like dyslipidemia were also reduced.

**FIGURE 6:**
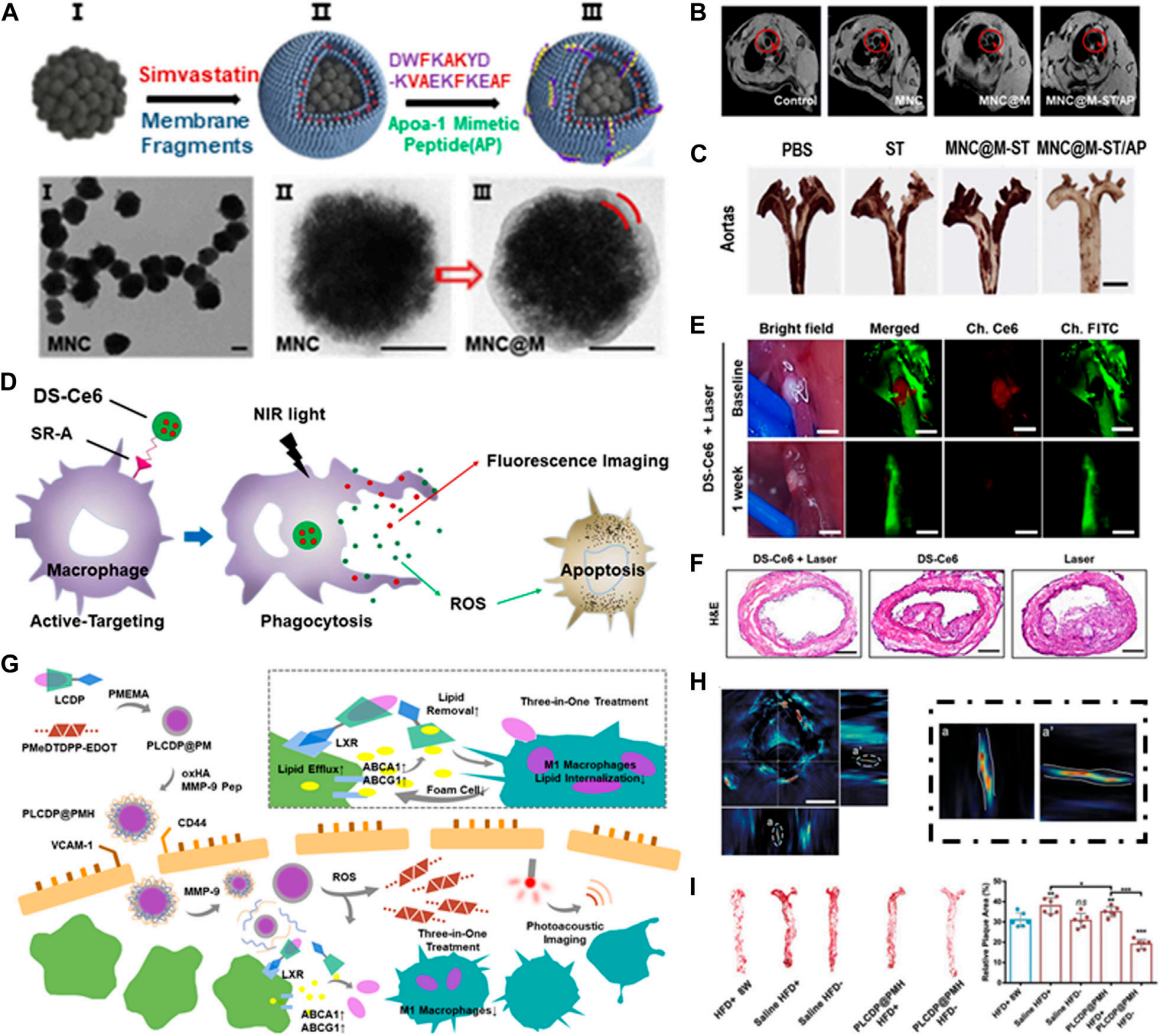
Integrated diagnosis and treatment of atherosclerosis. **(A)** Schematic illustration of the MNC@M-ST/AP fabrication. **(B)** *In vivo* MRI imaging of atherosclerotic plaque. **(C)** Photographs of excised aortas with ORO staining. Scale bar = 100 μm; Reprinted with permission (Wu et al., 2020). **(D)** Schematic illustration of the apoptosis-induction therapy. **(E)** *In vivo* imaging of the carotid atheroma at 48 h after the intravenous injection of DS-Ce6. Scale bar = 400 μm. **(F)** Histological validation of the therapeutic effects of DS-Ce6. Reprinted with permission (Song et al., 2021). **(G)** Schematic illustration of PLCDP@PMH theranostic nanoplatform. **(H)** Cross-section and side-looking photos of atherosclerotic plaque. **(I)** Photographs and relative area of the plaque after treatment. Reprinted with permission (Ma et al., 2022).

Wu et al. developed a self-driven nanovehicle, Fe_3_O_4_ magnetic nanoclusters (MNCs) was chosen as the core, and the surface was covered with leukocyte membrane fragments, anti-inflammatory drug Simvastatin (ST) and targetable apolipoprotein A-I mimetic 4F peptide (AP) were further loaded (Figure 6A) (Wu et al., 2020). The prepared biological nanovehicle (MNC@M-ST/AP) can be used for non-invasive MRI imaging (Figure 6B) and addresse the issue of hydrophobic drugs’ poor water solubility. After systematic administration, it can actively target to intimal macrophages in early atherosclerotic plaque, showing good anti-inflammatory and antioxidant effects. At the same time, the embedded AP can simultaneously suppress the uptake of oxLDL and promote cholesterol efflux, thus making the biologically activated nanocarriers a promising therapeutic system for the prevention of early atherosclerosis (Figure 6C).

Qiu et al. wrapped curcumin in superparamagnetic iron oxide nanoparticles (SPIO). The synthesized SDP-VCAM-1/Cur/Cy5.5 nanoparticles can not only be used as dual-imaging probes for MRI and fluorescence imaging, but also a carrier for delivering chemotherapy drugs to inflammatory tissues, providing a good opportunity for the molecular imaging and the targeted treatment of AS (Qiu and Xu, 2021).

### 5.2 Apoptosis-induction therapy

Song et al. developed a macrophage targeted near-infrared fluorescence (NIRF) emitting phototherapeutic agent DS-Ce6 by coupling dextran sulfate with Ce6 (Figure 6D). DS has high biocompatibility and biodegradability, and can target scavenger receptor (SR-A) highly expressed on macrophages and foam cells. Imaging guided DS-Ce6 light activation can detect inflammatory activity in atherosclerosis *in vivo* (Figure 6E). Photoactivation uses light of a specific wavelength to activate photosensitizers and converts oxygen into reactive oxygen species (ROS), such as singlet oxygen (^1^O_2_). ROS can kill inflammatory cells by inducing cell apoptosis and autophagy, thus reducing plaque burden and inflammation of atherosclerosis (Figure 6F) (Song et al., 2021).

Ye et al. described a nanoparticle combining the phase-transition material perfluorohexane (PFH) with dextran sulfate (DS). The results showed that the carrier can undergo phase transition under the irradiation of low intensity focused ultrasound (LIFU), which can induce the apoptosis of macrophage cells under ultrasound imaging, so as to achieve specific diagnosis and targeted treatment of vulnerable plaque (Ye et al., 2019).

Yang et al. constructed PFH@PLGA/MnFe2O4 Ram NPs, which can target vascular endothelial growth factor receptor2 (VEGFR2) and significantly induce apoptosis of neovascular endothelial cells after 3 days of photothermal (PTT) treatment. On the 28th day, PTT significantly reduced the density of new blood vessels and stabilized the plaque by inhibiting plaque hemorrhage and macrophage infiltration. In addition, PFH-mediated bubbles under thermal effect can enhance the resolution of US imaging (Yang et al., 2021). They further constructed PHPMR nanoparticles that can aggregate in the mitochondria of endothelial cells, and promoted mitochondrial caspase apoptosis through sonodynamic therapy (SDT), thereby reducing the density of new blood vessels and improving the stability of plaque (Yao et al., 2021).

Yao et al. proved that 5-aminolevulinic acid (ALA) mediated SDT can stabilize plaque by inducing the apoptosis and clearance of macrophage (Sun et al., 2019), and found sodium porphyrin (DVDMS) mediated SDT can induce endothelial cell apoptosis by reducing the level of VEGF-A in macrophage foam cells. The therapeutic effect was almost equivalent to that of 3-months statin treatment. They further conducted small-scale clinical trials in patients with atherosclerosis, suggesting a promising clinical strategy to prevent plaque hemorrhage (Yao et al., 2020).

### 5.3 Lipid-removal therapy

Although conventional anti-inflammation therapy had revealed certain beneficial efficiency, more reasonable designs are still required for thoroughly clearance of atherosclerosis. Ma et al. built a therapeutic nanoplatform combining ROS reactivity and two-photon AIE bioimaging for dimensional diagnosis and precise treatment of inflammation (Ma et al., 2020). The AIE fluorophore was bridged to β-cyclodextrin (CD) via a ROS-responsive bond, an anti-inflammatory drug prednisolone (Pred) was further carried by supramolecular interaction. The combination of CD and lipid was conducive to drug dissociation, so as to achieve effective lipid removal. The coating of RBC membrane can help nanoplatform achieve longer circulation time by escaping the phagocytic system (Ma et al., 2021). Similarly, they further constructed a multifunctional nanocarrier LFP-PCDPD, which utilized the high affinity of dextran to vascular adhesion molecule-1 (VCAM-1) and CD44 receptor on the surface of damaged ECs to achieve the active targeting of atherosclerosis (Hong Xu, 2022). Recently, they built a three-in-one nanopltform PLCDP@PMH (Ma et al., 2022). It can achieve integrated lipid management of reduced lipid-internalization, enhanced lipid-efflux and lipid-removal by inhibiting macrophage M1-polarization, the upregulation of ABCA1/G1, and cyclodextrin-assisted lipid dissolution (Figure 6G), in addition to photoacoustic imaging (Figure 6H), PLCDP@PMH can reverse the advanced atherosclerosis (Figure 6I), which provided a promising candidate for the diagnosis and treatment for atherosclerosis.

## 6 Discussion

In the past decades, the tremendous progress of imaging technology has strongly promoted the research from imaging the existed atherosclerotic plaque in symptomatic patients to characterizing the asymptomatic vulnerable plaque. It is worth noting that the application of nanotechnology in cardiovascular disease is certainly not limited to the injectable nanoparticles described in this review. However, atherosclerosis remains a major public health problem with devastating consequences. Nowadays, a large number of targeted nanoparticles have been constructed to achieve more imaging strategies for plaque characterization. By combining with the receptors of the surface, the nanoparticles can specifically gather in the atherosclerotic lesions, providing the expression or direct reading of the activity of fragile markers at the molecular level. This nanoparticle enhanced molecular imaging strategy may help to identify vulnerable plaque at early stage, and also help to determine which therapies are the best candidates for clinical trials.

Previous studies mainly focused on the targeting of atherosclerosis, and the nanoparticles were usually loaded with traditional AS medication prescriptions, such as rapamycin and statins. Although the therapeutic efficiency of drugs can be improved by targeting-delivery, the side effects still exist of these drug-dependent strategies. In recent years, researchers have developed a series of drug-independent therapies, including photothermal therapy and sonodynamic therapy, which can lead to apoptosis of AS components and thus lower the burden of plaque. The nanoparticle-mediated theranostic system, as a breakthrough in traditional medicine, enables nanoparticles to complete the diagnosis and therapy processes as a collective system. The exceptional performance of nanoparticle-mediated plaque imaging in preclinical studies will make the targeting accuracy of diagnostic reagents a crucial node for its continued development. On the one hand, the targeting of diagnostic agents can significantly increase the reliability of diagnosis. On the other hand, therapeutic agents can function at a lower concentration in the target region, greatly reducing the adverse effects of the theranostic system mediated by nanoparticles. Additionally, ensuring that diagnosis and therapy are carried out in unison will increase the precision of the nanoparticle-mediated theranostic system in terms of time, such as the activation of imaging and therapy under a single specific stimulus source.

Although the outstanding performance of nanoparticle-based diagnosis and treatment of plaque in preclinical experiments providing a new way for the management of atherosclerosis, clinically approved nanoparticles are still deficient, and the clinical transformation of nanoparticle-based imaging strategies in this field still faces great challenges: 1) Further histological research is needed to verify the accuracy of imaging; 2) The current research is to evaluate the progress of plaque by quantitatively evaluating the cumulative signals of nanoparticles at plaque, the diagnosis criteria should be determined before entering the clinical stage; 3) Unable to provide a complete pathophysiological assessment of whole coronary artery; 4) The biological behavior of nanoparticles, such as pharmacokinetics, targeting efficiency and biocompatibility, needs further research. It is expected that in the next few years, researchers will make full efforts to overcome these limitations of the existing design and make it widely used in clinical practice and research. Integrated intravascular imaging has gradually matured and is expected to become a favorable solution for atherosclerosis research and high-risk patient identification.
